# Physiological significance of pericoronary inflammation in epicardial functional stenosis and global coronary flow reserve

**DOI:** 10.1038/s41598-021-97849-5

**Published:** 2021-09-24

**Authors:** Yoshihisa Kanaji, Tomoyo Sugiyama, Masahiro Hoshino, Toru Misawa, Tatsuhiro Nagamine, Yumi Yasui, Kai Nogami, Hiroki Ueno, Hidenori Hirano, Masahiro Hada, Masao Yamaguchi, Rikuta Hamaya, Eisuke Usui, Taishi Yonetsu, Tetsuo Sasano, Tsunekazu Kakuta

**Affiliations:** 1 Division of Cardiovascular Medicine, Department of Cardiology, Tsuchiura Kyodo General Hospital, 4-4-1 Otsuno, Tsuchiura City, Ibaraki 300-0028 Japan; 2 Department of Cardiovascular Medicine, Tokyo Medical and Dental University, Tokyo, Japan

**Keywords:** Interventional cardiology, Atherosclerosis

## Abstract

Both fractional flow reserve (FFR) and global coronary flow reserve (g-CFR) provide prognostic information in patients with stable coronary artery disease (CAD). Inflammation plays a vital role in impaired endothelial dysfunction and atherosclerotic progression, potentially predicting cardiovascular mortality. This study aimed to evaluate the physiological significance of pericoronary adipose tissue inflammation assessed by CT attenuation (PCATA) in epicardial functional stenosis severity and g-CFR in patients with CAD. A total of 131 CAD patients with a single de novo epicardial coronary stenosis who underwent coronary CT-angiography (CCTA), phase-contrast cine-magnetic resonance imaging (PC-CMR) and FFR measurement were studied. PCATA was assessed using the mean CT attenuation value. G-CFR was obtained by quantifying absolute coronary sinus flow (ml/min/g) by PC-CMR at rest and during maximum hyperemia. Median FFR, g-CFR, and PCATA values were 0.75, 2.59, and − 71.3, respectively. Serum creatinine, NT-proBNP, left ventricular end-diastolic volume, and PCATA were independently associated with g-CFR. PCATA showed a significant incremental predictive efficacy for impaired g-CFR (< 2.0) when added to the clinical risk model. PCATA was significantly associated with g-CFR, independent of FFR. Our results suggest the pathophysiological mechanisms linking perivascular inflammation with g-CFR in CAD patients.

## Introduction

Inflammation plays an important role in the atherosclerotic progression and the rupture of coronary plaque, resulting in subsequent acute coronary syndrome^1–3^. A recent study reported that the pericoronary adipose tissue attenuation (PCATA) on coronary computed tomography angiography (CCTA) was associated with local inflammation and cardiac mortality^4^. Inflammation status of pericoronary adipose tissue detected by histology and ^18^F-fluorodeoxyglucose (FDG) uptake on positron emission tomography (PET) were significantly associated with PCATA^5^. For revascularization decision makings, fractional flow reserve (FFR) has rapidly gained a consensus as a gold standard of induced regional ischemia by epicardial coronary artery stenosis. FFR has been demonstrated to show a continuous and independent relationship with subsequent outcomes in patients with stable coronary artery disease (CAD)^6^. On the other hand, global coronary flow reserve (g-CFR) has been established as an integrated marker of the vasodilating capacity of the whole coronary artery system showing the powerful prognostic information, potentially linked with the microvascular function^7^. G-CFR obtained by PET also provides robust prognostic information in patients with CAD, independent of the presence or absence of obstructive atherosclerotic coronary lesions^7–9^. Phase-contrast cine-magnetic resonance imaging (PC-CMR) allows non-invasive quantification of myocardial blood flow (MBF) and g-CFR by quantifying coronary sinus flow (CSF) without need for ionizing radiation, radioactive tracers, gadolinium, or intravascular catheterization, which have been validated against PET^10,11^.

Until today, the relationship between FFR, g-CFR, and PCATA, particularly, the impact of PCATA, the pericoronary inflammatory measures, on regional and global physiological measure are yet to be determined. Furthermore, the incremental capability of PCATA for predicting impaired g-CFR, when added to the clinical model, including FFR, remains unknown. Thus, in the present study, we tested the hypothesis that PCATA was significantly associated with both FFR and g-CFR. We further evaluated if PCATA showed the incremental discriminatory efficacy to predict impaired g-CFR when added to the prediction model including FFR. To test this hypothesis, the present study was undertaken by measuring FFR, g-CFR, and PCATA in CAD patients with a single epicardial de novo lesion and preserved systolic function.

## Methods

**Figure 1:**
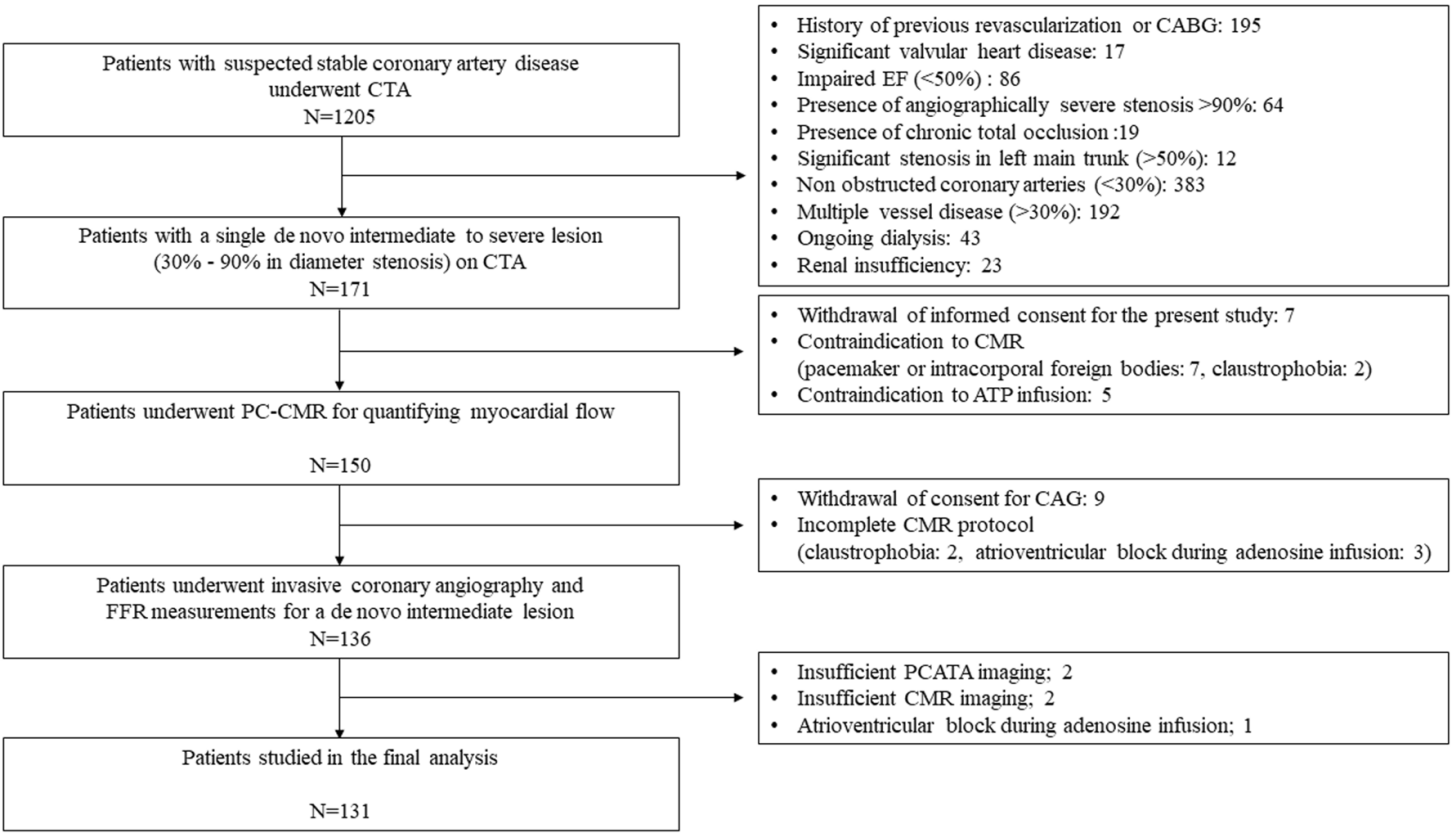
Study flow chart. Figure shows the screening and enrollment process with a total of 131 patients in the final analysis.

In this retrospective study, we enrolled consecutive patients with a single de novo intermediate to severe stenosis (30–90% by visual estimation) by clinically indicated CCTA for suspected CAD and subsequently underwent PC-CMR examination prior to diagnostic coronary angiography (CAG) within 60 days at Tsuchiura Kyodo General Hospital (Fig. 1). A total of 1205 patients with low to intermediate risk of obstructive coronary artery disease underwent CCTA from January 2018 to December 2019. CCTA indication was in accordance with a scientific statement from the American Heart Association Committee on Cardiovascular Imaging and Intervention^12^ and finally decided at the physician’s discretion. Patients with an intermediate to severe de novo lesion (30–90% in diameter stenosis) in a single vessel on CCTA who gave the written informed consent were enrolled and underwent PC-CMR study for quantifying coronary sinus flow and g-CFR. Patients without stenosis more than 30% on CCTA, with severe stenosis (> 90%) or chronic total occlusion, significant stenosis in the left main trunk, and multiple obstructed vessels (> 30% in diameter stenosis) were excluded. In the remaining 150 patients, 136 patients with written consent underwent invasive coronary angiography and subsequent FFR measurements for the lesions. Inclusion criteria included the following: age > 20 years and the detection of an identifiable, de novo lesion located in the proximal or mid-portion of a native coronary artery. Stable coronary artery disease was defined by consistent frequency, duration, or intensity of anginal symptoms within the 6 weeks before CAG. Exclusion criteria included previous coronary artery bypass surgery or percutaneous coronary intervention (PCI), ongoing dialysis, renal insufficiency with a baseline serum creatinine level > 1.5 mg/dl, angiographically significant left main coronary artery disease, culprit lesion of the acute coronary syndrome, an occluded culprit lesion, multiple vessel disease, visible collateral flow and contraindication to CMR (e.g., pacemaker, internal defibrillator or other incompatible intracorporeal foreign bodies, pregnancy, and claustrophobia). Patients with impaired systolic ejection fractions (< 50%) were also excluded. The study protocol agreed with the Declaration of Helsinki and was approved by the institutional ethics committee of Tsuchiura Kyodo General Hospital. All patients provided written informed consent before enrollment in this study.

### Cardiac catheterization and physiological assessment

Each patient initially underwent standard selective coronary angiography via the radial artery using a 5-F or 6-F catheter system. Coronary angiograms were analyzed quantitatively using a QAngio XA system (Medis Medical Imaging Systems, Leiden, The Netherlands). FFR was measured during stable hyperemia induced by intravenous adenosine (140 μg/kg/min through a central vein). These measurements were performed as part of the diagnostic catheterization, and the patients with FFR values ≤ 0.80 underwent ad-hoc PCI.

### Coronary CT-angiography acquisition

CCTA was performed using a 320-slice CT scanner (Aquilion ONE; Canon Medical Systems Corporation, Otawara, Tochigi, Japan) in accordance with the society of cardiovascular computed tomography guidelines^13^. Details are described in Method 1 in the Supplemental Materials.

### Analysis of PCATA

**Figure 2:**
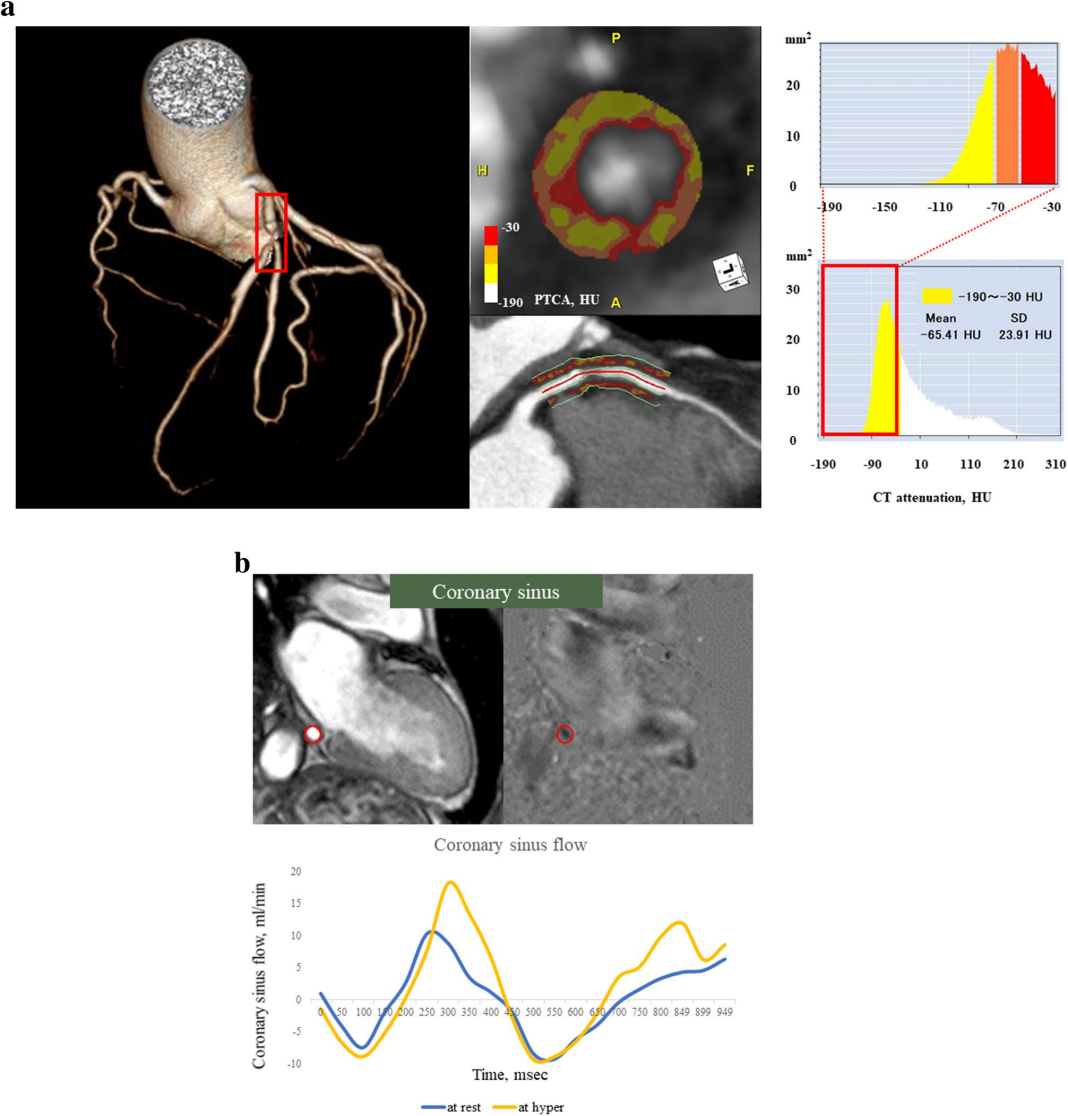
Representative case images. (**a**) Coronary computed tomography angiography image of pericoronary adipose tissue attenuation and a representative coronary sinus flow measurement. Pericoronary adipose tissue attenuation is defined as the mean CT attenuation value (− 190 to − 30 Hounsfield units [HU]) within a radial distance equal to the diameter of the vessel. (**b**) Phase-contrast cine-magnetic resonance images of the coronary sinus flow measurement. The proximal coronary sinus was detected in cross-section on the magnitude and phase-contrast images. The coronary sinus blood flow curves (Blue line: resting flow; Yellow line; hyperemic flow) were generated.

In the present study, the crude analysis of PCATA of all three main coronary vessels was performed. The mean PCATA of three main coronary vessels was used for the analysis. PCATA analysis was performed in the proximal 40 mm segments of left anterior descending coronary artery and left circumflex coronary artery and the proximal 10 to 50 mm segment of the right coronary artery using a dedicated workstation (Aquarius iNtuition Edition version 4.4.13; TeraRecon Inc., Foster City, CA, USA), as previously described^4,5^. Within the pre-identified segment of interest, the lumen as well as the inner and outer vessel wall border were tracked in an automated manner with additional manual optimization. Adipose tissue was defined as all voxels with attenuation between − 190 HU and − 30 HU. The PCATA was defined as the average CT attenuation in HU of the adipose tissue located within a radial distance from the outer vessel wall equal to the diameter of the coronary vessel (Fig. 2a). PCATA analysis was separately performed as a post hoc analysis blinded to the baseline characteristics and PC-CMR results at the institutional imaging and physiology lab by the expert investigator for PCATA analysis.

### CT-derived LV mass index and cardiac mass at risk

LV mass was indexed by body surface area (LV mass/BSA; LVMI). The % cardiac mass at risk was defined as the % ratio of the subtended cardiac mass at risk to the whole left ventricular myocardial mass. Details are described in Method 2 in the Supplemental Materials.

### CMR image acquisition, coronary sinus flow and g-CFR measurement

CMR image acquisition was performed using a 1.5-Tesla scanner (Philips Achieva, Philips Medical Systems, Best, The Netherlands) with 32-channel cardiac coils. PC-CMR image acquisition and g-CFR quantification by absolute CSF measurement were performed as previously described^10,14,15^. Briefly, the coronary sinus was identified in the atrioventricular groove using the basal slices of the short-axis stack. The plane for flow measurement by PC-CMR was positioned perpendicular to the CS at 1 to 2 cm from the ostium^10^. PC-CMR of the CSF measurement was performed during maximal hyperemia and at rest. Maximal stable hyperemia was induced by intravenous adenosine (140 μg/kg per min through a central vein). The CSF quantitative analyses were performed in a blinded fashion by two expert investigators (Y. Kanaji and T Misawa), using proprietary software (Philips View Forum, Best, The Netherlands) (Fig. 2b). The resting CSF value was corrected using rate pressure products (RPP) as follows^10,14^; rate pressure product = systolic blood pressure (mm Hg) × heart rate; corrected CSF = (CSF/RPP) × 10,000; and corrected CSF (ml/min per g) = corrected CSF/LVM (g). G-CFR was evaluated by CSF reserve, which was calculated as CSF during maximal hyperemia divided by resting CSF.

### Statistical analysis

The patients were divided into two groups by the g-CFR value of 2.0, which are indicated to be associated with major cardiac adverse events^7,8^. Clinical characteristics, CCTA-derived data, and CMR-derived variables were compared between these two groups. Statistical analyses were performed using SPSS version 25.0 (IBM Corporation, Armonk, NY, USA). Categorical data were expressed as numbers and percentages and compared by chi-square or Fisher’s exact tests, as appropriate. Continuous data were expressed as median (interquartile range [IQR]) and analyzed using the Mann–Whitney test and the variance for variables with non-normal distribution and normal distribution, respectively. Correlations between 2 variables were assessed using Pearson’s correlation analysis. Receiver operating characteristic curves were analyzed to assess the best cutoff values for predicting g-CFR < 2.0 (impaired g-CFR). Univariable and multivariable linear regression analyses were performed to determine predictive factors of FFR and g-CFR (stepwise-forward method; P < 0.05). Univariable and multivariable logistic regression analyses were also performed to predict g-CFR < 2.0. The Hosmer–Lemeshow statistic was applied to assess model calibration. The prediction model for g-CFR < 2.0 was constructed to determine the incremental discriminatory and reclassification performance of mean PCATA by using relative integrated discrimination improvement (IDI) and category-free net reclassification index (NRI). A 2-tailed value of P < 0.05 was considered statistically significant.

## Results

### Patient characteristics

**Figure 3:**
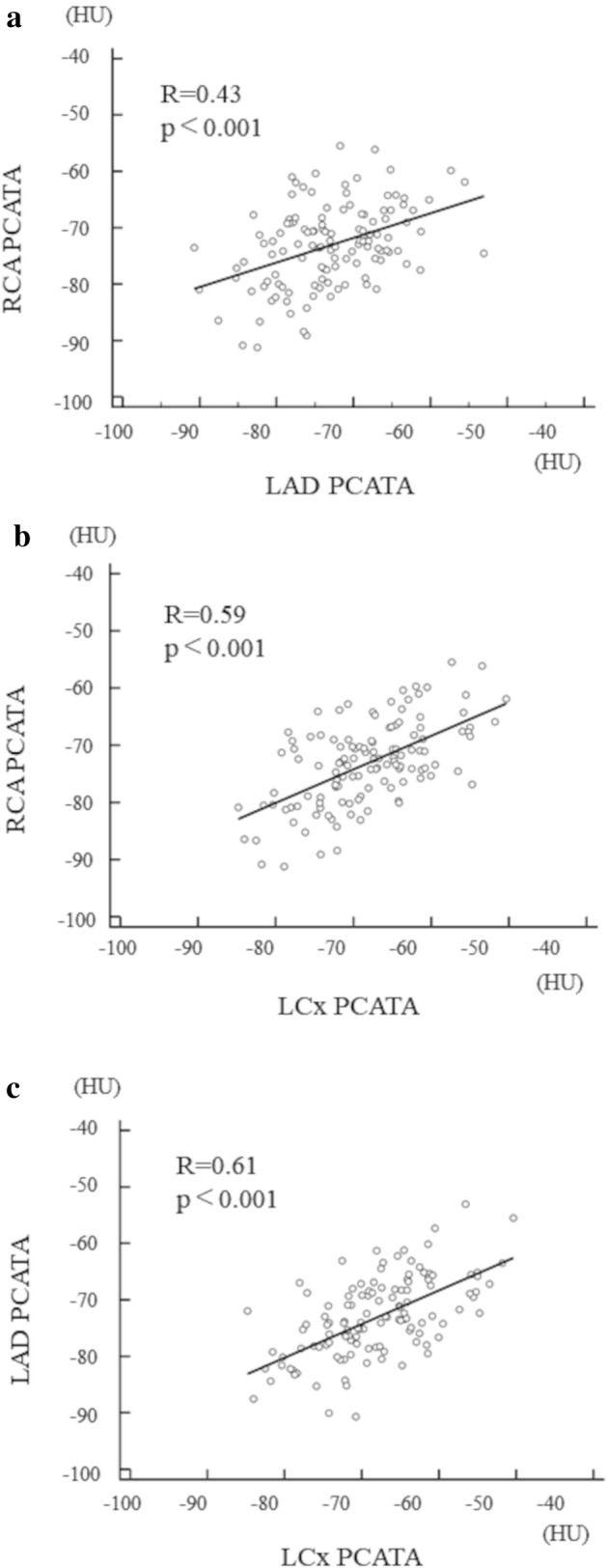
Association of PCATA values between the three major epicardial coronary arteries. (**a**) RCA and LAD, (**b**) RCA and LCx, and (**c**) LAD and LCx.

The final analysis was performed by 131 patients in the present study (Fig. 1). The mean time interval between CCTA, CMR, and CAG was 45 days (28–52 days). The patients’ baseline characteristics in the two groups divided by the presence or absence of impaired g-CFR (< 2.0) are summarized in Table 1. Patient demographics, laboratory data including hs-CRP, angiographic stenosis severity, and lesion locations were comparable between the two groups except for creatinine level. The median FFR value was 0.75 and similar between the two groups. Although LVM and LVMI were both significantly different, subtended cardiac mass at risk by the target lesion was not significantly different between the groups. Significant mutual relationships among RCA, LAD, and LCx PCATA values were observed (Fig. 3), which was in line with the previous report^4^. Mean PCATA of 3 major vessels and target vessel PCATA were higher in the group with impaired g-CFR compared with those without (P < 0.001, P = 0.003, respectively). Baseline CSF was significantly greater, and hyperemic CSF was significantly lower, resulting in significantly lower g-CFR values in the impaired g-CFR group. No significant association was observed between PCATA and hs-CRP (P = 0.30).

**Table 1:** Clinical characteristics of patients with and without impaired g-CFR (g-CSF < 2.0).

	Total N = 131	With impaired g-CFR N = 43	Without impaired g-CFR N = 88	P value
**Demographics**				
Age, year	67 ± 10	68 ± 11	67 ± 9	0.83
Male, n (%)	87 (66.4)	33 (76.7)	54 (61.4)	0.08
Body surface area, m^2^	1.70 [1.56, 1.83]	1.73 [1.60, 1.84]	1.68 [1.55, 1.83]	0.46
**Medical history, n (%)**				
Hypertension, n (%)	97 (74.0)	31 (72.1)	66 (75.0)	0.72
Hyperlipidemia, n (%)	84 (64.1)	26 (60.5)	58 (65.9)	0.54
Diabetes mellitus, n (%)	55 (42.0)	16 (37.2)	39 (44.3)	0.44
Current smoker, n (%)	35 (26.7)	10 (23.3)	25 (28.4)	0.53
Family history, n (%)	20 (15.3)	6 (14.0)	14 (15.9)	0.77
**Prescription at admission, n (%)**				
Statin, n (%)	114 (87.0)	36 (83.7)	78 (88.6)	0.43
ACE-I or ARB, n (%)	75 (57.3)	26 (60.5)	49 (55.7)	0.60
β-blocker, n (%)	49 (37.4)	20 (46.5)	29 (33.0)	0.13
Calcium antagonist, n (%)	62 (47.3)	18 (41.9)	44 (50.0)	0.38
**Coronary angiography**				
Target lesion location; RCA/LAD/LCx, n (%)	20 (15.3)/103(78.6)/8(6.1)	8(18.6)/33(76.7)/2(4.7)	12 (13.6)/70(79.5)/6(6.8)	0.70
MLD, mm	0.85 [0.67, 1.26]	0.82 [0.69, 1.30]	0.85 [0.66, 1.19]	0.55
RD, mm	2.82 [2.43, 3.17]	2.82 [2.34, 3.34]	2.83 [2.49, 3.11]	0.67
DS, %	66.9 [56.8, 76.1]	66.8 [57.0, 75.7]	68.1 [56.8, 76.4]	0.67
Lesion length, mm	13.0 [10.1, 18.0]	13.0 [10.1, 16.9]	13.3 [10.1, 18.5]	0.99
**Physiological data**				
FFR	0.75 [0.61, 0.79]	0.73 [0.63, 0.79]	0.77 [0.58, 0.80]	0.52
FFR ≤ 0.75, n (%)	67 (51.1)	24 (55.8)	43 (48.9)	0.46
**Laboratory data**				
T-chol, mg/dl	181 [154, 208]	181 [144, 213]	180 [156, 205]	0.98
LDL-chol, mg/dl	97 [78, 125]	95 [76, 130]	98 [79, 123]	0.85
HDL-chol, mg/dl	50 [44, 60]	48 [42, 54]	52 [44, 62]	0.10
TG, mg/dl	125 [91, 182]	135 [79, 176]	125 [93, 197]	0.70
Creatinine, mg/dl	0.82 [0.70, 0.93]	0.83 [0.75, 0.95]	0.80 [0.66, 0.90]	0.035
eGFR, ml/min 1.73/m^2^	69.5 [60.1, 77.6]	70.1 [57.6, 76.5]	69.2 [61.4, 80.0]	0.43
HbA1c, %	6.0 [5.6, 6.9]	6.0 [5.5, 7.0]	6.1 [5.7, 6.9]	0.54
NT-proBNP, ng/l	92.0 [38.5, 208.3]	116.0 [49.8, 289.3]	74.5 [37.0, 198.5]	0.11
cTnI at presentation, ng/l	4.0 [2.0, 9.8]	3.0 [2.0, 9.0]	4.0 [2.0, 10.0]	0.90
hs-CRP, mg/dl	0.070 [0.030, 0.155]	0.080 [0.030, 0.130]	0.065 [0.030, 0.160]	0.59
**CT data**				
Whole LV mass volume, cm^3^	136.5 [115.6, 160.3]	141.4 [123.1, 171.2]	133.0 [108.2, 157.5]	0.011
Whole LV mass volume, g	144.0 [121.9, 169.1]	149.2 [129.8, 180.6]	140.3 [114.1, 166.2]	0.011
LV mass index by CT, g/m^2^	83.4 [73.4, 93.3]	85.9 [77.3, 97.3]	82.4 [70.8, 91.7]	0.015
Area at risk mass volume, %	32.7 [24.0, 39.0]	30.7 [22.4, 38.5]	33.0 [25.0, 39.0]	0.60
Area at risk mass volume, cm^3^	43.5 [31.1, 54.4]	45.7 [31.4, 55.6]	42.1 [30.1, 53.5]	0.21
Area at risk mass volume, g	45.9 [32.8, 57.4]	48.2 [33.2, 58.6]	44.4 [31.8, 56.4]	0.21
Agatston score (target vessel)	135.0 [25.1–385.7]	214.6 [59.2–382.0]	104.8 [18.3–390.2]	0.20
Agatston score (total)	314.0 [66.7–790.2]	487.5 [118.5–914.3]	180.2 [45.4–775.1]	0.065
Mean PCATA	− 71.3 [− 75.9, − 67.9]	− 68.2 [− 72.0, − 65.4]	− 72.8 [− 77.5, − 69.4]	< 0.001
Highest PCATA in major 3 vessels	− 67.4 [− 71.4, − 62.4]	− 63.4 [− 66.4, − 60.9]	− 69.2 [− 72.0, − 64.9]	< 0.001
Target vessel PCATA	− 73.4 [− 77.9, − 67.9]	− 69.5 [− 75.4, − 65.2]	− 74.1 [− 78.5, − 69.5]	0.003
RCA PCATA	− 73.2 [− 78.8, − 69.0]	− 70.8 [− 74.5, − 66.6]	− 73.6 [− 80.1, − 70.4]	0.019
LAD PCATA	− 73.8 [− 78.2, − 68.2]	− 70.2 [− 75.4, − 65.2]	− 75.0 [− 79.3, − 69.3]	0.001
LCx PCATA	− 69.1 [− 72.7, − 63.8]	− 64.2 [− 69.6, − 61.1]	− 70.7 [− 74.3, − 65.2]	< 0.001
**CMR indices**				
EDV, ml	105.6 [92.4, 121.9]	117.2 [100.1, 130.3]	102.8 [91.8, 118.7]	0.018
ESV, ml	33.9 [29.2, 43.8]	36.0 [30.8, 47.7]	33.0 [27.7, 43.4]	0.10
EF, %	65.8 [60.2, 71.0]	65.0 [60.1, 71.0]	66.3 [61.6, 70.9]	0.57
CSF at rest, ml/min	119.8 [88.9, 162.8]	149.2 [113.0, 181.1]	107.9 [81.9, 150.7]	0.001
CSF at rest, ml/min/g	0.97 [0.67, 1.23]	1.01 [0.80, 1.27]	0.93 [0.65, 1.20]	0.12
Corrected CSF at rest, ml/min	128.3 [95.0, 168.2]	161.0 [128.8, 203.1]	110.5 [89.2, 145.2]	< 0.001
Corrected CSF at rest, ml/min/g	0.99 [0.79, 1.26]	1.08 [0.92, 1.65]	0.91 [0.73, 1.19]	0.004
CSF at hyperemia, ml/min	316.0 [238.8, 388.8]	247.9 [183.5, 315.7]	349.7 [279.7, 423.8]	< 0.001
CSF at hyperemia, ml/min/g	2.44 [1.84, 3.16]	1.87 [1.26, 2.29]	2.70 [2.23, 3.63]	< 0.001
g-CFR	2.59 [1.92, 3.37]	1.78 [1.36, 1.99]	3.13 [2.55, 3.74]	< 0.001
Corrected g-CFR	2.39 [1.88, 3.22]	1.62 [1.20, 1.87]	2.89 [2.37, 3.53]	< 0.001

*ACE-I* angiotensin-converting enzyme inhibitor, *ARB* angiotensin receptor blocker, *cTnI* cardiac troponin I, *CT* computed tomography, *CSF* coronary sinus flow, *EDV* end diastolic volume, *EF*, ejection fraction, *eGFR* estimated glomerular filtration rate, *ESV* end systolic volume, *g-CFR* global coronary flow reserve, *HbA1c* glycated hemoglobin, *HDL-chol* high density lipoprotein cholesterol, *hs-CRP* high sense c-reactive protein, *HU* Hounsfield units, *LAD* left anterior descending coronary artery, *LCx* left circumflex coronary artery, *LDL-chol* low density lipoprotein cholesterol, *LVM* left ventricular mass, *LVMI* left ventricular mass index, *NT-proBNP* N-terminal pro-B-type natriuretic peptide, *PCATA* pericoronary adipose tissue attenuation, *RCA* right coronary artery, *TG* triglyceride, *TIMI* thrombolysis in myocardial infarction.

### Determinants of FFR

The univariable and multiple linear regression analyses were performed to examine the factors associated with FFR. (Table 2) The multivariable analysis demonstrated that minimum lumen diameter (MLD) (P < 0.001) and target vessel PCATA or the mean PCATA of 3 major vessels were independent predictors of FFR (P < 0.001, P < 0.001, respectively). There was a significant and robust relationship between the target vessel PCATA and the mean PCATA of 3 major vessels (R = 0.79, P < 0.001). The target vessel PCATA and the mean PCATA showed similar relationships with FFR, although the numerical difference in the strength of the relationship was observed. (Table 2).

**Table 2:** Univariate and multiple linear regression analysis for FFR.

	Univariable analysis			Multivariable analysis 1			Multivariable analysis 2		
	β	95% CI	P value	β	95% CI	P value	β	95% CI	P value
Age, year	0.002	0.000 to 0.004	0.10	0	− 0.001 to 0.003	0.43	0.001	− 0.001 to 0.003	0.47
Male	− 0.053	− 0.100 to − 0.005	0.03	0.023	− 0.020 to 0.065	0.29	0.031	− 0.012 to 0.007	0.68
MLD, mm	0.163	0.121 to 0.205	< 0.001	0.165	0.124 to 0.206	< 0.001	0.168	0.129 to 0.207	< 0.001
DS, %	0	− 0.001 to 0.000	0.24						
Lesion length, mm	− 0.001	− 0.004 to 0.002	0.61						
NT-proBNP, pg/ml	5.3 × 10^–6^	0.000 to 0.000	0.68						
cTnI at presentation, ng/l	0	− 0.002 to 0.001	0.83						
hs-CRP, mg/dl	0.19	− 0.019 to 0.058	0.33						
Target lesion: LAD	0.084	0.030 to 0.138	0.003	0.030	− 0.019 to 0.078	0.23	0.042	− 0.004 to 0.089	0.077
LV mass index by CT, g/m^2^	− 0.001	− 0.002 to 0.001	0.28						
Area at risk mass volume, g	− 0.001	− 0.002 to 0.000	0.026	0	− 0.019 to 0.078	0.12	− 0.001	− 0.002 to 0.000	0.082
Agatston score (Target vessel)	− 2.1 × 10^−5^	0.000 to 0.000	0.43						
EF, %	0.001	− 0.002 to 0.004	0.62						
Mean PCATA	− 0.007	− 0.010 to − 0.003	< 0.001			Not selected	− 0.007	− 0.010 to − 0.003	< 0.001
Highest PCATA	− 0.007	− 0.010 to − 0.003	< 0.001			Not selected			Not selected
Target vessel PCATA	− 0.006	− 0.009 to − 0.003	< 0.001	− 0.005	− 0.008 to − 0.003	< 0.001			Not selected
CSF at hyperemia, ml/min/g	0.004	− 0.015 to 0.023	0.67						
Corrected g-CFR	0.009	− 0.012 to 0.031	0.39						

### Determinants of g-CFR

**Figure 4:**
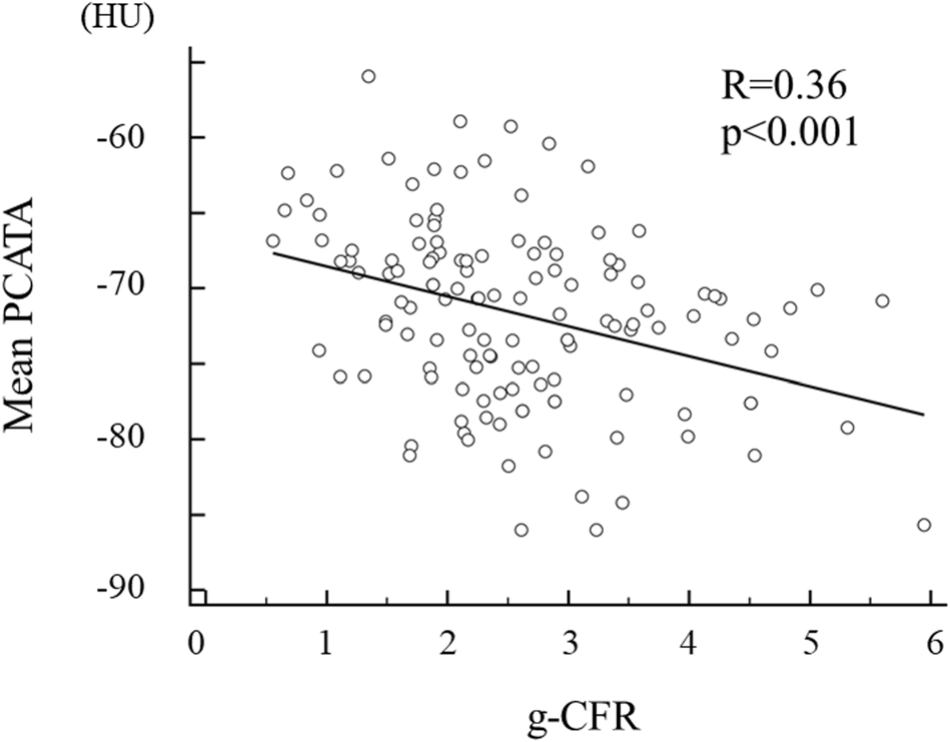
A linear relationship between mean PCATA and g-CFR. There is a significant relationship between mean PCATA and g-CFR (R = 0.37, P < 0.001).

To examine the factors associated with g-CFR, we performed univariable and multiple linear regression analyses (Table 3). The univariable analysis identified that creatinine level, log (NT-proBNP), LV mass volume, LVMI, subtended cardiac mass volume at risk, end-diastolic left ventricular volume (EDV), target vessel PCATA, and the mean PCATA remained as significant factors. On multivariable analysis, creatinine level, log (NT-proBNP), EDV, and mean PCATA were significant factors to predict g-CFR (P = 0.024, P = 0.030, P = 0.041, and P = 0.004 respectively). The linear relationship between pre-PCI PCATA and g-CFR was shown in Fig. 4. On multivariable logistic regression analysis, independent predictors of g-CFR < 2.0 were creatinine level (OR 9.482, 95% CI 1.019–88.223, P = 0.048) and mean PCATA (OR 1.141, 95% CI 1.060–1.229, P < 0.001). The Hosmer and Lemeshow test provided P values of 0.735, which indicated proper goodness of fit for this model (Table 4).

**Table 3:** Univariate and multiple linear regression analysis for g-CFR.

	Univariable analysis			Multivariable analysis 1			Multivariable analysis 2		
	β	95% CI	P value	β	95% CI	P value	β	95% CI	P value
Age, year	− 0.006	− 0.026 to 0.013	0.54						
Male	− 0.235	− 0.629 to 0.159	0.24						
HDL-chol, mg/dl	0.003	− 0.013 to 0.020	0.69						
Creatinine, mg/dl	− 1.162	− 2.018 to − 0.306	0.008	− 1.064	− 1.873 to − 0.255	0.010	− 0.917	− 1.710 to − 0.123	0.024
cTnI at presentation, ng/l	− 0.009	− 0.022 to 0.004	0.18						
log (NT-proBNP), pg/ml	− 0.172	− 0.321 to − 0.023	0.024	− 0.088	− 0.240 to 0.064	0.29	− 0.152	0.289 to − 0.015	0.030
CRP, mg/dl	0.176	− 0.142 to 0.494	0.28						
FFR	0.624	− 0.796 to 2.045	0.39						
Whole LV mass volume, g	− 0.009	− 0.014 to − 0.004	< 0.001						
LV mass index by CT, g/m^2^	− 0.017	− 0.026 to − 0.008	< 0.001				− 0.004	− 0.014 to 0.007	0.50
Area at risk mass volume, g	− 0.010	− 0.018 to − 0.002	0.019				0	− 0.001 to 0.008	0.85
Mean PCATA	− 0.061	− 0.090 to − 0.032	< 0.001			Not selected	− 0.049	− 0.081 to − 0.016	0.004
Highest PCATA	− 0.048	− 0.074 to − 0.023	< 0.001			Not selected			Not selected
Target vessel PCATA	− 0.040	− 0.065 to − 0.014	0.002	− 0.019	− 0.047 to 0.008	0.17			Not selected
EDV, ml	− 0.012	− 0.020 to − 0.005	0.001	− 0.008	− 0.016 to 0.001	0.004	− 0.008	− 0.015 to 0.000	0.041

**Table 4:** Univariate and multivariate logistic regression analysis for factors to predict impaired g-CFR (g-CFR < 2.0).

	Univariable analysis			Multivariable analysis 1			Multivariable analysis 2		
	HR	95% CI	P value	HR	95% CI	P value	HR	95% CI	P value
Age, year	1.004	0.967–1.043	0.83						
Male	2.078	0.908–4.753	0.083						
HDL-chol, mg/dl	0.979	0.946–1.013	0.21						
Creatinine, mg/dl	11.182	1.190–105.102	0.035	10.614	1.036–108.771	0.047	9.903	1.375–127.6	0.047
cTnI at presentation, ng/l	1.011	0.986–1.036	0.39						
log (NT-proBNP), pg/ml	1.270	0.942–1.712	0.12						
CRP, mg/dl	0.769	0.358–1.651	0.50						
FFR	0.627	0.040–9.910	0.74						
FFR ≤ 0.75	1.322	0.635–2.751	0.46						
Whole LV mass volume, g	1.015	1.004–1.026	0.006						
LV mass index by CT, g/m^2^	1.030	1.009–1.051	0.004	1.018	0.995–1.042	0.052	1.007	0.987–1.029	0.070
Area at risk mass volume, g	1.012	0.9955–1.030	0.16						
Mean PCATA	1.140	1.060–1.225	< 0.001			Not selected	1.141	1.060–1.229	0.002
Highest PCATA	1.116	1.049–1.188	0.001			Not selected			Not selected
Target vessel PCATA	1.096	1.034–1.161	0.002	1.095	1.032–1.161	0.003			Not selected
EDV, ml	1.020	1.004–1.036	0.016	1.011	0.993–1.029	0.10	1.011	0.994–1.030	0.19

### Incremental discriminatory and reclassification performance of PCATA

Clinical risk model 1 was constructed by using age, male sex, diabetes mellitus, creatinine, high sense troponin I, NT-proBNP, LVMI, target lesion in LAD, and area at risk of subtended cardiac mass volume. NRI and IDI indices were significantly improved when PCATA was added to the clinical risk model 1 + FFR for predicting g-CFR < 2.0, whereas FFR showed no significant additive predictive information to the clinical risk model 1 (Table 5).

**Table 5:** Comparison of discriminant and reclassification ability of clinical models.

Prediction model	AUC (95% CI)	P value	IDI	P value	NRI	P value
Clinical model 1	0.69 (0.60–0.78)		Reference		Reference	
Clinical model 2: model1 + FFR	0.69 (0.59–0.78)	0.81	0.0013	0.66	0.0655	0.72
Clinical model 3: model 2 + mean PCATA	0.79 (0.70–0.87)	0.015	0.0936	< 0.001	0.4604	0.003
Clinical model 2: model1 + FFR	0.69 (0.59–0.78)		Reference		Reference	
Clinical model 3: model 2 + mean PCATA	0.79 (0.70–0.87)	0.015	0.0923	< 0.001	0.5307	0.003

To determine incremental discriminatory and reclassification capacities of mean PCATCA for predicting g-CFR < 2.0

Clinical model 1: age, male sex, DM, creatinine, cTnI at presentation, log (NT-proBNP), LV mass index, target lesion: LAD, Area at risk.

## Discussion

The important findings of the present study are as follows. In patients with CAD with a single de novo stenosis and preserved systolic function; (1) the mean PCATA was significantly associated with epicardial functional stenosis severity determined by FFR; (2) the mean PCATA was an independent predictor of reduced g-CFR. To the best of our knowledge, this is the first study to demonstrate the physiological significance of pericoronary inflammation evaluated by CT attenuation in epicardial functional stenosis severity and g-CFR.

### FFR and PCATA

Inflammation has been widely demonstrated to play a pivotal role in driving atherosclerotic progression. The results of the present study indicated that PCATA was significantly associated with FFR. It has been recently reported that high-risk atherosclerotic plaque morphology was significantly associated with FFR^16^. Perivascular inflammation drives the development and progression of coronary atherosclerosis^5^. PCATA, a surrogate marker of local inflammation, was demonstrated to be associated with coronary plaque instability and high-risk characteristics^17^. Our result is in line with these studies. Although current findings may provide an important insight into the association between perivascular inflammation and epicardial functional stenosis severity, other factors such as lesion morphology, plaque burden, plaque specific inflammation, local oxidative stress, and endothelial dysfunction might affect stenosis severity as previously reported^18,19^, further large population studies are required to test this hypothesis-generating finding.

### G-CFR and PCATA

G-CFR has been demonstrated as a powerful predictor of worse outcomes independent of epicardial coronary stenosis severity^7,8,20^. However, pathophysiologic determinants of global CFR have not been fully elucidated. Our main hypothesis of the present study was to evaluate the physiological significance of PCATA in epicardial functional stenosis severity and g-CFR. Considering the complexity and difficulty of evaluating the association of epicardial functional stenosis with global flow by the limited population, we focused on the single de novo lesion with intermediate to severe stenosis in the present study, by excluding multivessel disease and including functionally significant and non-significant stenoses in the study population. The present study exhibited a relatively weak albeit statistically significant association between pericoronary adipose tissue inflammation and g-CFR. This relationship was independent of FFR and hs-CRP, which were used as standard markers of regional ischemia and systemic inflammation. Our findings are in line with the previous study by Taqueti et al.^8^ in which g-CFR obtained by PET was associated with outcomes regardless of the angiographic severity of coronary artery disease, although our study could not evaluate outcomes. G-CFR is an integrated marker of coronary function, including epicardial coronary stenosis, diffuse atherosclerosis, and microvascular dysfunction^21^. Our results may suggest that patients with intermediate to severe epicardial coronary disease have a significant extent of diffuse disease and/or microvascular dysfunction, impacting on g-CFR independent of and over the regional ischemia in association with pericoronary inflammation^22^. There is growing evidence that microvascular dysfunction is associated with increased inflammation and may precede or coexist with high-risk coronary atherosclerosis^22,23^. In addition, it has also been demonstrated that endothelial dysfunction and coronary microvascular dysfunction could be affected by systemic inflammation^3,24^. Our results further indicate the importance of the link between inflammation and microvascular dysfunction because both may share a similar characteristic that extends beyond coronary vascular territory, not confined to a target vessel territory, which is defined by FFR. Our findings suggest that not only epicardial lesion severity, but also other factors including microvascular function, endothelial function and vasodilatory ability may be linked with the extent of pericoronary inflammation in patients with coronary artery disease and preserved systolic function. This hypothesis is merely a speculative explanation of the mechanism linking local perivascular adipose tissue inflammation with g-CFR and our results showed no causal inference between perivascular inflammation and atherosclerosis. The present study included only the patients with single-vessel disease. Assuming that the severity of diffuse atherosclerosis or microvascular disease in non-obstructive vessels might play an important role in the association between PCATA and reduced or preserved g-CFR in patients with CAD, further studies are needed to clarify if the sum of FFR values of three major coronary arteries significantly correlated with global CFR and/or PCATA. Global CFR represents not only the effect of epicardial stenosis but the integral of the coronary artery flow characteristics including diffuse non-obstructive disease and microvascular dysfunction. Considering the relatively weak albeit statistically significant association between PCATA and g-CFR, other factors such as renal impairment linking with oxidative stress and endothelial inflammation might play an important role in the reduction of g-CFR^25^. Mechanistic insights of reduced global CFR may be multi-factorial and complex, thus further large sample size studies are needed to define the pathophysiological mechanisms and outcomes linking with inflammation and g-CFR. Incremental prognostication by these variables over clinical risk factors should be also evaluated in the future studies.

### Clinical implications of PCATA

Our results indicated that low-level local inflammation, which was not associated with hs-CRP, could be assessed by PCATA, and showed a significant association with both FFR and g-CFR. In contrast, FFR did not contribute to g-CFR values in CAD patients with single epicardial stenosis and preserved systolic function. Considering that global CFR has been established as an integrated marker of the pathophysiological status of epicardial coronary stenosis, diffuse atherosclerosis, microvascular function, and myocardial tissue perfusion^21^, the disease monitoring and patient managements might be guided by the combined assessment of PCATA and g-CFR using CT and CMR that are widely available and accessible in the clinical practice, since the association between PCATA and g-CFR in this study is relatively weak. The present study, however, could evaluate no causal inference between PCATA and g-CFR. Recently, van Diemen et al. reported that RCA PCATA was a significant predictor of worse outcomes in CAD patients, independent of myocardial ischemia^26^. The authors evaluated regional hyperemic myocardial blood flow (MBF), and hyperemic MBF of each vessel did not correlate with vessel-specific PCATAs. In contrast, we analyzed the mean PCATA which may represent the net assessment of 3 vessel specific PCATA obtained from 3 major coronary arteries, indicating the status of inflammation of the whole heart pericoronary adipose tissue. Our results that the regional functional stenosis evaluated by FFR was not correlated with g-CFR were in line with their study, although our study had no outcome data. Given that no significant relationship was observed between FFR and g-CFR, these two crucial indices evaluate different pathophysiological abnormalities in CAD patients and might provide complementarily prognostic information. Our result demonstrated that NT-pro BNP and serum creatinine level were the significant factors of impaired g-CFR. Coronary microvascular dysfunction has been proposed to be an important mechanism underlying the pathogenesis of heart failure, while previous study showed that comorbidities such as obesity, diabetes mellitus, chronic kidney disease led to systemic inflammation, coronary endothelial inflammation and microvascular dysfunction^27^. The results of this study suggest that pericoronary inflammation obtained by CCTA may provide the assessment of diverse pathophysiologic pathways responsible for the development of atherosclerosis in CAD and might help identify high-risk CAD patients for worse outcomes independent of regional functional stenosis. Furthermore, future therapeutic strategies directed towards reducing PCATA that represent the specific inflammatory status of the cardiovascular system may potentially provide a novel management option for improving prognosis.

## Study limitations

The results of the present study should be interpreted with consideration for several significant limitations. First, this study included a relatively small number of patients from a single-center, which may not allow extensive subgroup analysis or more reliable multivariable analyses. Furthermore, this study was conducted on the limited population with single-vessel disease. The future studies including multi-vessel disease are crucial since multi-vessel CAD has been reported to be observed in 40–50% of stable coronary artery disease (CAD) patients and are correlated with worse clinical outcomes compared with single-vessel CAD in clinical practice^28^. Second, this study provided no outcome data. Third, CMR perfusion imaging was not performed according to the protocol in the present study. CMR perfusion-defined ischemia might provide an important insight into the association between PCATA and regional CFR. Forth, currently, there is no widely available proprietary software that can automatically analyze PCATA. However, the software used in the present study is commercially available, and the CT hardware used was a single system that gives us the strength of the study. Furthermore, CT is currently an essential tool to risk-stratify patients with known and suspected coronary artery disease with worldwide availability^29,30^. Forth, in this study, the use of statins was about 90%, but LDL levels were still suboptimal (median 97 mg/dl). Statins have been reported to provide a significant reduction of vascular inflammation^31^. Although further high dose statins might have reduced PCATA, it is plausible that residual inflammation after lipid-lowering therapy was suggested from our results of PCATA, because no significant relationship between LDL levels and PCATA was observed and the non-negligible portion of patients with LDL levels lower than 70 mg/dl showed increased PCATA. In other words, targeting PCATA after lipid-lowering therapy may provide the opportunity to manage residual risk. Finally, our study is cross-sectional and merely hypothesis-generating; thus, it cannot discern the temporal relationship between pericoronary inflammation and reduced g-CFR. Further studies are needed to test our results.

## Conclusions

The present study demonstrated the significant relationship between pericoronary inflammation represented by PCATA and g-CFR independent of epicardial stenosis severity evaluated by FFR in CAD patients with a single de novo lesion and preserved systolic function. PCATA might be able to monitor disease extent or test the impact of future therapeutic interventions. Further studies with larger sample sizes and outcome measures are warranted.

## Supplementary Information

Supplementary Information.

## Data Availability

The datasets used and/or analyzed during the current study are available from the corresponding author on reasonable request.
