# Supplementary material for: Physiological significance of pericoronary inflammation in epicardial functional stenosis and global coronary flow reserve
Source: Sci Rep. 2021 Sep 24;11:19026. doi: 10.1038/s41598-021-97849-5 (PMC8463533; doi:10.1038/s41598-021-97849-5)
Supplement: Supplementary file 1 — Supplementary Information. [file 41598_2021_97849_MOESM1_ESM.docx]

**Supplemental materials**

**Method 1.** *Coronary CTA acquisition*

When needed, oral and/or intravenous beta-blockers were administered to achieve a target heart rate ≤ 65 bpm. A non-contrast enhanced CT-scan for the assessment of coronary artery calcification, prospectively triggered at 75% of the RR-interval with 3 mm slice thickness, was followed by CT angiography. Immediately before CTA scanning, 0.3 or 0.6mg of sublingual nitroglycerine was administered. The scan was triggered using an automatic bolus-tracking technique with a region of interest placed in the ascending aorta. Images were acquired after a bolus injection of 40-60 mL contrast (iopamidol, Bayer Yakuhin, Ltd., Japan) at a rate of 3–6mL/s, using prospective ECG-triggering or retrospective ECG gating with tube current modulation. Acquisition and reconstruction parameters for the patients in our study were 120 kVp, tube current of 50 to 750mA, the gantry rotation speed of 350 ms per rotation, helical pitch of 8-18, field matrix of 512x512, and scan thickness of 0.5 mm. All scans were performed during a single breath-hold. Images were reconstructed at a window centered at 75% of the R-R interval to coincide with left ventricular diastasis.

**Method 2.** *CT-derived LV Mass Index and Cardiac Mass at Risk*

Quantitative assessments of LV mass were performed using the Aquarius iNtuition Workstation Edition version 4.4.13 (TeraRecon Inc., Foster City, CA, USA) at mid-diastole phase as previously described (1). The cardiac mass was calculated as the left ventricular myocardial volume derived by the manually corrected automated delineation of the epicardial and endocardial contours and multiplied with the specific gravity of myocardial tissue (x1.055gr/mL). Papillary muscles were not included in the calculation of LV mass. Coronary artery–based myocardial segmentation was performed to evaluate a coronary lesion-specific cardiac mass by using the same dedicated software (Aquarius iNtuition Edition version 4.4.13; TeraRecon Inc., Foster City, CA, USA) by the expert investigator blinded to the clinical, angiographic, and physiological data as a post hoc analysis. The coronary tree and left ventricular myocardium were extracted semi-automatically, and the cardiac mass at risk was defined as the myocardial mass subtended distal to the culprit lesion identified by using CCTA. The myocardial territories of the 3 coronary arteries and subtended myocardium by the functionally significant stenotic lesion were assigned using the 3-dimensional Voronoi algorithm.

1. Fuchs A et al. Normal values of left ventricular mass and cardiac chamber volumes assessed by 320-detector computed tomography angiography in the Copenhagen General Population Study. Eur Heart J Cardiovasc Imaging 2016;17:1009-17.
